# State of the art on lung organoids in mammals

**DOI:** 10.1186/s13567-021-00946-6

**Published:** 2021-06-02

**Authors:** Fabienne Archer, Alexandra Bobet-Erny, Maryline Gomes

**Affiliations:** UMR754, IVPC, INRAE, EPHE, Univ Lyon, Université Claude Bernard Lyon 1, 69007 Lyon, France

**Keywords:** Lung, Organoid, Stem cells, 3D culture, Infection, In vitro model

## Abstract

The number and severity of diseases affecting lung development and adult respiratory function have stimulated great interest in developing new in vitro models to study lung in different species. Recent breakthroughs in 3-dimensional (3D) organoid cultures have led to new physiological in vitro models that better mimic the lung than conventional 2D cultures. Lung organoids simulate multiple aspects of the real organ, making them promising and useful models for studying organ development, function and disease (infection, cancer, genetic disease). Due to their dynamics in culture, they can serve as a sustainable source of functional cells (biobanking) and be manipulated genetically. Given the differences between species regarding developmental kinetics, the maturation of the lung at birth, the distribution of the different cell populations along the respiratory tract and species barriers for infectious diseases, there is a need for species-specific lung models capable of mimicking mammal lungs as they are of great interest for animal health and production, following the One Health approach. This paper reviews the latest developments in the growing field of lung organoids.

## Introduction

Organoids are three-dimensional (3D) multicellular in vitro self-assembled constructs that mimic the corresponding in vivo organ in terms of cell type, structure and function. They are generated from stem cells or progenitors in a process that simulates the molecular and cellular stages of organ development. They are currently developed to mimic multiple organs including the mammary gland, intestine and stomach, liver, kidney, blood vessels, brain, pancreas and lung [1]. In the lung, damage to or disruption of the epithelium that covers the entire respiratory tract can lead to respiratory failure, inflammation or the development of tumors or disabling chronic diseases. Understanding the cellular and molecular mechanisms controlling lung morphogenesis and function provides the framework for understanding its development, its regeneration and the pathogenesis of acute and chronic lung diseases. Animal models have been instrumental in providing understanding of lung development and diseases. However, given the differences between species, there is a need for species-targeted lung models, and although the need for human models is of great importance regarding human health, we also need to develop models that mimic other mammals which are of great interest for animal health and production, by following the One Health approach.

## The complex architecture and cell composition of the lung

The lung is a complex organ composed of semi-rigid conducting tubes that branch and narrow from the trachea, bronchi and bronchioles, leading to highly vascularized saccules or alveoli, where respiratory gases are exchanged. The airways are composed of several types of cells primarily resulting from the embryonic neuroectoderm, mesoderm and endoderm. In the mature lung, a wide variety of cell types are found in specific proportions and locations that create the architectural elements on which ventilation depends. This complex structure is protected from continuous exposure to particles, pathogens, and toxicants by the secretion of protective mucus and surfactant, active mucociliary clearance and by a robust innate and acquired immune system. This unique architecture of the mammalian lung is required for adaptation to breathing air at birth and thereafter [2].

In order to accommodate diverse functions, the lung possesses several specialized cell types [2, 3]. The conducting airways are mainly populated by four epithelial cell types: ciliated, goblet, Club cells and basal cells [4]. Ciliated cells have multiple cilia that beat in a synchronous rhythm in order to maintain the flow of mucus across the airway epithelium. Goblet cells are secretory cells that secrete mucus on the surface of the airways. The basal cells are the adult stem cells of the airways. In humans, basal cells line the main bronchi and large bronchioles, but begin to decrease in number in the smaller bronchioles toward the alveoli. Other sparse populations of cells in the airway include club cells, which are secretory cells that secrete lubricating glycosaminoglycans and antimicrobial peptides into the airways, and pulmonary neuroendocrine cells which are innervated on the basal surface and store proteins that are released under a physiological stimulus such as hypoxia. Rare other cell types have been described in the airways, such as ionocytes, pulmonary neuroendocrine cells (PNECs), brush cells and a population of NREP-positive cells [4].

The alveolar epithelium is composed of two epithelial cell types, alveolar epithelial type I cells (AECI) and alveolar epithelial type II cells (AECII). AECIs form a thin, squamous epithelium that covers the majority of the alveolar surface and exchanges gas with neighboring capillaries through diffusion. AECIIs have a cuboidal shape and secrete surfactant proteins to reduce the surface tension of the alveolar sacs, allowing them to expand and contract without collapsing as breathing takes place.

It is noteworthy that lung structure and composition is highly species dependent [5, 6]. In most species the respiratory system develops through prenatal and postnatal life, and there are considerable interspecies differences in the timing and duration of the different steps of their development [7–11]. One of the most interesting points regarding the subject of this paper, is that the cells lining the bronchiolar or more distal portion of the tracheobronchial tree, vary significantly in terms of abundance, the types of cell present, the ultrastructural features of these cells in adult animals, and the secretory products that they produce [12, 13]. For example, in humans, the basal cells containing pseudostratified epithelium extend distally to terminal bronchioles of about 0.5 mm in diameter, and only the respiratory bronchioles are lined by a simple cuboidal epithelium lacking basal cells. By contrast, a pseudostratified epithelium is largely restricted to the mouse trachea, and the transition to a simple columnar epithelium without basal cells occurs in the mainstem bronchi. Thus, the cellular composition and organization of the mouse intrapulmonary airway epithelium essentially resembles only the most distal portions of human conducting airways, whereas the mouse trachea is much more similar to most of the airway generations of the human lung [14]. Another illustration is the difference in composition of ciliated cells: the human respiratory trachea present more ciliated cells (49%) than horse (46%), cow (42%), pig (43%) and sheep (31%) [13]. Another interesting point concerns the age of maturation of the lung, and more precisely the stage of alveolar development at birth which varies quite considerably between species; in mouse and rat it is largely postnatal, whereas in guinea pig, sheep and humans it begins in utero and is relatively advanced at birth [15]. In sheep, lung maturation proceeds rostro-caudally, with the apical of the lobes maturing much earlier than the basal lobes. Most of what we know about lung homeostasis is mainly inferred from rodent studies [2] and to some extent from ovine lungs [16]. Indeed, while the mouse model is traditionally considered the preferred model to mimic the human lung, due to its cost-effectiveness and ease of genetic manipulation, it has its own drawbacks such as the over-representation of Club cells along the airways, late postnatal lung maturation, lack of metastasis and complex analysis of the immune response. For many years, the sheep model has shown interesting proximities to the human lung in terms of similar anatomy, comparable distribution of differentiated cells, and similar maturation age. These different elements make the sheep a valuable model for human respiratory physiology and diseases, such as surfactant studies in preterm infants/lambs, cancer development, asthma, cystic fibrosis and viral infection [17–21].

## Endogenous stem/progenitor cells of the lung

The adult lung is a highly quiescent tissue with a slow turn-over rate (< 1% per day) of airway and alveolar epithelia [22]. However, following tissue damage, lung has shown an extraordinary regenerative capacity to repair tissue damage and to restore its functions. Although previously based on morphological and ultrastructural studies, the description of the diversity and the complex lineage of the specific stem and progenitor lung cells is becoming increasingly more detailed with advances in cell lineage tracing, flow cytometric analyses, single cell RNA sequencing methods, proteomics, transcriptomics and progress in imaging. Numerous reviews have described the different lung stem cells identified, mainly characterized in humans and mice [15, 22–25]. Descriptions of lung stem cells in other medium and large mammals are still very sparse, except in our work on the identification of ovine bronchioloalveolar progenitors with dual differentiation potential [26].

The proximal airway contains basal stem cells (Trp63+, Krt5+) as progenitors, that are able to self-renew and give rise to secretory (SCGB1a1+), ciliated and neuroendocrine cells [27–29]. In addition to basal cells, several other cells can respond to injury by gaining in plasticity to act as progenitors and contribute to homeostasis by proliferating and repopulating to replace lost cells. Secretory cells are able to generate new ciliated cells after injury [30].

Similar to the proximal airway, the distal airways and alveolar regions contain both putative, dedicated stem cells and a population of cells that are able to transdifferentiate to regenerate the epithelial layers after injury. At least two subpopulations of bronchiolar Scgb1a1-expressing cells, Clara cells, and vClara cells have been described as progenitors [31]*.*

In the mouse, bronchio-alveolar stem cells (BASC) reside at the junction between the distal airway and the alveolus and can give rise to multiple cell types upon injury such as club cells (Scgb1a1+) or AECII (Sftpc+) [32]. However, it is still unclear whether human or other mammal lungs contain BASCs. Additionally, in both the mouse and the human, AECII cells in the alveoli have been shown to act as an alveolar progenitor, proliferating to replenish lost AECII cells after injury, and giving rise to AECI cells in mice [33–35]. Interestingly, AECI cells have also been reported to give rise to AECII cells in the mouse after injury [36]. Recent evidence suggests that there are also rare stem/progenitor cells in the distal lung that do not express mature lineage markers [lineage negative epithelial progenitors (LNEP)] and that become active in response to severe distal lung injury in mice [37]. Using a single-cell RNA sequencing approach, Travaglini et al. [38] recently proposed a molecular cell atlas of the human lung by which they defined the gene expression profile and anatomical location of not less than 58 different lung cell populations. This atlas could provide the foundation for more in-depth investigation of the varieties of existing lung cells, their functions in development, regeneration and their behavior in responses to disease.

## Mimicking the lung in vitro

Mimicking the lung in vitro is a rather difficult challenge as it entails: (i) reproducing the complex diversity of airways and different alveolar cell types, (ii) /reproducing specific functions that mimic the upper airway respiratory protection, by secreting specific mucus to filter and trap external materials and by actively removing these trapped materials via an active ciliary beating activity, and (iii) mimicking the alveolar space where essential gas exchanges occur through a thin layer of epithelial cells towards the vascular system.

To date the vast majority of in vitro lung models are developed from mouse and human tissues, with protocols published, describing the method used to isolate and cultivate primary adult lung epithelial cells alone or in coculture, as monolayers, as a polarized epithelium (2D) or at the air–liquid interface (ALI) [39]. Few data are available on other model species, especially those of agronomic interest, but we can point to several papers on porcine [40], bovine [41–44], equine [45] and ovine [26, 46] lung cells. Primary adult differentiated lung epithelial cells remain difficult to isolate due to their low turn-over. They rapidly lose their in vivo phenotype, they can exhibit high variability in cell composition from one isolation or animal to another, and can often only be expanded by dedifferentiation. On the other hand, the few available lung epithelial immortalized cell lines hardly differentiate or retain an epithelial relevant phenotype. Although the precision cut lung slices approach has gained much interest in the study of specific lung diseases (cancer, infection), slices are difficult to maintain in vitro on a long term basis and it is still necessary to slaughter an animal for each set of experiments. From the ethical and societal standpoint, the development of alternative methods to animal experiments is now a necessity. New alternative methods must be able to satisfy one or more principles of the 3Rs rules: replacing (substitution of animal models whenever possible), reduction (reducing the number of animals in experimentation), and refinement (optimizing the methodology applied to animals). Organoids can overcome the current lack of relevant and efficient in vitro lung models, by taking advantage of stem cell plasticity. Indeed, many of the limitations cited above can be overcome by generating organoids from progenitors, pluripotent stem cells, embryonic stem cells or induced pluripotent stem cells (iPS) that are readily expandable.

### Organoids derived from embryonic stem cells or induced pluripotent stem cells

One of the first descriptions of lung organoid culture was published by Zimmermann in 1987 [47]. By isolating and cultivating cells from mice fetuses, the author was able to generate an organoid structure, with the formation of a lumen in the center of the organized aggregates, cell differentiation towards alveolar AECII (presence of lamellar bodies) and the production of a matrix by the mesenchymal cells surrounding organoids. Although these first experiments opened the door to deriving lung organoids from different species in vitro, the process is still complex and requires mimicking specific steps of differentiation with the activation or inactivation of key signaling pathways regulating developmental patterning in lengthy, sequenced and precise protocols. Indeed, the greatest progress in generating lung organoids has been made by basing the differentiation protocols on the cascade of activated signaling pathways that direct embryonic lung development [48, 49]. Step-by-step approaches were successfully established for the differentiation of embryonic stem cells (ESCs) into functional airways and alveolar cells [50, 51]. In the first step of stem cell induction, Activin-A, which belongs to the transforming growth factor β (TGFβ) signaling, plays the key role in activating the germ-layer specification of the endoderm. Then, the main following pathway involved in lung organoid establishment involves Wnt and FGF as effectors of the differentiation, and BMP and TGF as repressors. On top of this, an essential element for obtaining the 3D organization of the cells as organoids, is the culture of the cells into a gelatinous matrix (embedding) resulting from the basement membrane extract, which will physically participate in the architecture of the structure and molecularly in cell maturation.

Induced pluripotent stem cells (iPSCs) are the product of adult somatic cells that are reprogrammed into an embryonic-like state (OCT4+, SOX2+, SSEA4+, TRA1-60+), and their utilization has become an effective strategy as a new source of stem cells and for developing patient-specific lung epithelial cells. As for ESCs, and in order to drive iPSCs into the lung epithelium, it is necessary to follow a stepwise differentiation protocol to generate definitive endoderm (DE) cells (CXCR4+, SOX17+, FOXA2+), then anterior foregut endoderm (AFE) cells (NKX2.1+, PAX9+, SOX2+) and subsequently, to obtain populations of basal, goblet, ciliated, club cells, AECI, and AECII cells. Huang et al. reported an optimized method for generating FOXA2+NKX2.1+ progenitors from human definitive endoderm cells at an efficiency rate of 86% [52]. These progenitors were shown to give rise to basal, club, goblet, ciliated, AECI, and AECII cells both in vivo and in vitro. Human iPSC-derived AECII cells exhibit a self-renewal capacity and could display immune responsiveness [53–55].

**Figure 1:**
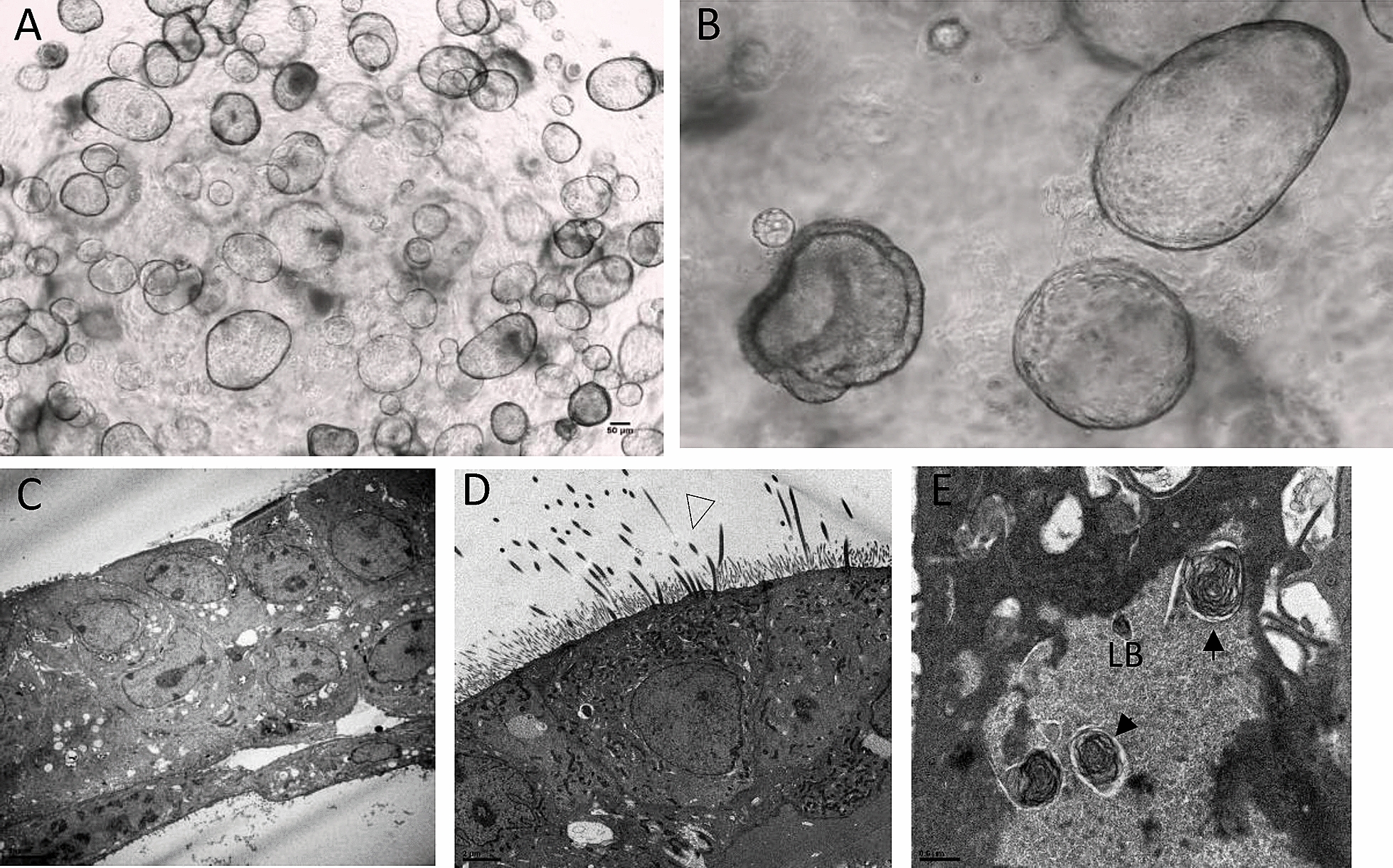
**Bovine lung organoids issued from embryonic lungs**. **A**, **B** organoids are cultivated and amplified in Matrigel^R.^. They are composed of **C** various cell types, such as **D** ciliated cells of the bronchi and **E** AECII cells from the alveoli, with typical lamellar bodies (transmission electron microscopy).

Taken together, these protocols give rise to organoids composed of proximal and/or distal pulmonary epithelial populations that can be developed over the long term, amplified, frozen and exposed to different stimuli and analyzed [3, 4, 56–59]. As mentioned above, almost all of the protocols were developed and tested using human and mice models. Presently, no examples of lung organoids from domestic animals using pluripotent stem cells have been published. Based on protocols for human lung organoids as previously listed, we succeeded in deriving bovine lung organoids from embryonic lungs (unpublished results), taken at different stages of maturation (Figure 1). These organoids exhibited ciliary activity and surfactant production, demonstrating the presence of airway and alveolar mature epithelial cells.

### Organoids derived from adult stem cells

To overcome the complexity and cumbersome protocols for the differentiation of pluripotent stem cells (ESCs or iPSCs), new approaches have been developed from adult tissues, taking advantage of the potential of self-renewal and differentiation of tissue-resident adult stem cells. Among all the epithelial cells lining the respiratory tract, basal cells (tracheobronchial region), club cells and bronchioloalveolar stem cells-BASCs (Brionchiolar region), and AECII cells (Alveolar regions) have shown their capacity to generate organoids in mice and humans (for a review see [56, 60]). Protocols vary with different medium compositions (specific growth hormones, activators or inhibitors of specific pathways), the differential isolation step of specific stem cell/progenitors, coculture with or without stromal cells, and the possible first step of culture in 2D conditions with an air–liquid interface (ALI) before culture in the matrix. The most important factors involved in the generation of lung organoids from adult stem cells involves WNT and FGF as activated pathways, and ROCK, MAPK, TGF and BMP as repressed pathways. More recently, Salahudeen et al. [61] developed a robust feeder-free, chemically-defined culture of distal human lung progenitors as organoids derived clonally from single adult human AECII or KRT5 positive basal cells. AECII organoids exhibited AECI transdifferentiation potential, while basal cell organoids progressively developed lumens lined by differentiated club and ciliated cells. In addition to lung tissue removed surgically, organoids could be generated from the culture of lung epithelial cells collected from bronchoalveolar lavage fluid (BALF) [62].

## Lung organoids for what?

### Biobanking

Cultured organoids may serve as a sustainable source of functional cells. Indeed, lung organoids derived from pluripotent stem cells (hPSCs), including ESC and iPSC, have the potential to make a powerful impact on our understanding and treatment of lung disease in different species. Efforts are currently underway to generate populations of immature lung epithelial and mesenchymal progenitors that can be massively expanded—with the option of storage in a cryobank—and then directed to differentiate efficiently into mature airway and/or alveolar tissues [63]. Generating personalized organoids would also open novel avenues of research into individual responses to therapies, and thus also for the implementation of personalized medicine.

Although the molecular mechanisms that drive lung development and repair are likely to be conserved in general between mice and humans, work with early human embryos has already revealed interspecies differences in the transcription factors and ligands that are crucial at certain stages. Therefore, it will be important to examine the expression and function of specific genes in the lung in relevant models when addressing domestic animals.

### Disease modeling

Lung organoids are important resources for modeling diseases. They can be useful for studying genetic pulmonary diseases and lung cancer, and screening appropriate drugs and therapies [64–67]. Cystic fibrosis is a relatively common genetic disease worldwide and is associated with a loss of CFTR gene function (numerous mutations reported), encoding a chloride channel regulating the mucosal environment. It has been shown that airway organoids from patients with cystic fibrosis recapitulate central disease features, with reduced forskolin-induced swelling and response to CFTR modulators VX-770 and VX-809 [62]. Another important lung disease that can be modeled with organoids is idiopathic pulmonary fibrosis IPF. This disease is characterized by progressive fibrotic scarring in the lung tissue surrounding the air sacs, ultimately leading to dyspnea. TGF-β is upregulated and activated in IPF and modulates fibroblast phenotype and function in the lung. Using 3D lung organoids from patients with IPF, Surolia et al*.* observed that inhibiting the assembly of vimentin intermediate filaments reduced the invasiveness of lung fibroblasts in the majority of the subjects tested [68].

Lung cancer remains the most commonly diagnosed cancer and the leading cause of cancer death worldwide. Organoids established from human lung cancer resections and metastasis biopsies retain tumor histopathology as well as cancer gene mutations and are amenable to drug screening [62, 69]. Sachs et al. have shown that individual tumor alveolar organoids (AOs) vary greatly in their respective responses in line with their mutational profile. Regarding their specific and individual mutation for p53, ERBB2 and ALK1, the different AOs were more or less sensitive to treatment with the p53-stabilizing drug Nutlin-3a, to EGFR/ERBB2 and to ALK/ROS inhibitors, respectively [62]. These different examples demonstrate that organoids can recapitulate lung dysfunctions and tumor histology in vitro, and serve as platforms to screen drugs and molecular therapeutic correctives approaches.

### Genetic modifications

The possibility of deriving pulmonary organoids from different species, and from different individuals, whether healthy or carriers of a genetic disease, opens up the possibility of molecular corrective therapeutic approaches. The first demonstration of functional repair of an organoid was performed by targeting a defective receptor, the cystic fibrosis transmembrane conductor receptor (CFTR), which is associated with cystic fibrosis disease. The authors used the CRISPR/Cas9 genome editing system to correct the CFTR locus by homologous recombination in the intestinal organoids of CF patients [70]. More recently, the Xu’s team demonstrated that gene correction using CRISPR/Cas9 tool, could restore CFTR function in iPSC-derived proximal lung organoid cells [71]. Using CRISPR/-Cas9 to introduce frameshift mutations in Hermansky–Pudlak syndrome (HPS) genes, Strikoudis et al. generated human ESC-derived lung organoids that presented fibrotic changes, mimicking IPF and thus providing a platform for identifying pathogenic mechanisms of this disease [66].

### Regenerative medicine

One long‐term goal of organoid technology may be in regenerative medicine. An initial approach could benefit transplantation as cultured organoids could be used as a sustainable source of functional cells, but several hurdles remain to be overcome (safety of the cells, capacity to generate neoplasms, efficient protocols, etc.). In the shorter term, better comprehension of the molecular mechanisms driving lung development and stem cell activation and differentiation could help to solve the imbalances in lung cell composition that are observed, for example, in smokers and in Chronic Obstructive diseases (COPD) that present hyperplasia of basal cells. Targeting or inhibiting specific pathways such as Hedgehog, Notch, and retinoic acid could help to control the balance between basal and luminal cells, and increase number of ciliated cells at the expense of club cells [25]. Lung organoids could help to validate the efficacy of these therapies and restore balanced lung function.

### Infectious diseases

Respiratory diseases have a very high impact on human and animal health. Moreover, these diseases are among the most economically important diseases affecting cattle on a worldwide basis. Mainly due to respiratory infections, they result in poor animal welfare, economic losses and increased antibiotic consumption. The lungs are constantly exposed to the external environment and the infectious and toxic agents present in the air. Both viral and bacterial pathogens trigger damage to the lung epithelial cells, leading to the alteration of respiratory efficiency and in some cases to severe illness of the animal affected. Those threats represent key health and economic issues for cattle, including bovine tuberculosis and bronchopneumonia, two major pathologies. These bovine diseases have their human counterparts, namely bronchiolitis in infants and human tuberculosis. Tuberculosis remains a great health threat to the global population, with nearly 10 million new mycobacterium tuberculosis infections reported annually over the past 5 years, according to a WHO TB report. Other pathogens such as Influenza viruses can strongly impact human and/or animal health, by altering the airway and alveolar epithelium. Faced with constant environmental modifications and evolutions of pathogens, better understanding of the physio-pathological processes of infection remains one of the main goals for controlling, preventing and treating these diseases. Better knowledge of how pathogens target and interact with specific population of lung cells is necessary to fight them more efficiently. Moreover, being able to predict the pathogenicity of different circulating or emerging strains of viruses and bacteria could help to develop preventive and curative measures.

Human and medium/large mammal models are preferable to mice models when studying infectious disease pathogenesis, because pathogens often have a narrow species or tissue tropism; that is, they infect only certain species, and sometimes only specific cell types. Lung organoids have shown their suitability as models for studying respiratory viruses. Human parainfluenza virus (HPIV) and respiratory syncytial virus (RSV) were shown to successfully infect human airway organoids [72] and, in the case of RSV, to cause dramatic changes in epithelial morphology and function, with increased motility of airway epithelial cells [62]. Differentiated airway organoids were used to predict the infectivity of emerging respiratory viruses such as human and avian influenza viruses [73–75] and are currently under investigation for emerging zoonotic coronaviruses such as SARS-CoV-2 [61, 76–78]. Given the crucial capacity of some respiratory pathogens to induce devastating pandemics in farm animals and, in the case of certain of them to pass on to humans and induce major zoonosis, developing species specific respiratory organoids will help to better understand and predict animal and human infectious diseases. These complex cellular platforms could be used to screen therapeutic drugs as well as anti-microbial drugs [62].

If the field of respiratory infectious diseases, virologists are taking increasing advantage of organoid models as platforms to decipher the mechanisms of viral infection, cell deregulation and drug screening/development, but there is still much to do regarding bacterial [79], and parasitic infections. Mention can be made of a recent study from Heo et al., who took advantage of organoids to elucidate the interaction of a human protozoan parasite, Cryptosporidium, with intestinal and lung epithelia, the two main sites of infection [80]. Studies of parasites, including the development of effective treatments, are often hampered by the lack of a robust in vitro system that recapitulates the life cycle in the host. Authors have shown that after injection of Cryptosporidium oocysts into the organoid lumen, the parasite propagates within the organoids and completes its complex life cycle. Using a transcriptomic approach, they highlighted the importance of interferon-I signaling in response to Cryptosporidium infection [80].

We expect in the future that more respiratory pathogens or co-cultures of pathogens, completed at some point with lung microbiota (commensal and symbiotic microorganisms), will be explored, as it is currently being done in gastrointestinal organoids [81, 82].

## The future of lung in vitro models

Progress is being made in establishing organoid cultures in which multiple cell types (stromal, endothelial, immune, nerve cells) are combined, and this will help to establish how cell types interact in vivo and how they are affected by injury, inflammation, infection and aging (see for example [83, 84]). However, progress in lung organoid models will require the identification of more surface markers and/or reporters for isolating and purifying subpopulations of stem and progenitor cells and stromal support cells.

It is also likely that extracellular matrix components and physical forces play key roles in regulating stem cell behavior and differentiation, and these parameters are also beginning to be explored using organoid systems. The extracellular matrix (ECM) in the native lung is intricate and complex, and shapes the cell microenvironment by providing structural cues, regulating access to soluble growth factors and controlling cell adhesion and migration. In an effort to create a hospitable environment for human lung cells to thrive, researchers have turned to bioengineering approaches to create a variety of structural scaffolds (Matrigel, collagen, synthetic material such as PLGA…) and microenvironments to reproducibly mimic the lung [83].

To model the mechanical forces of the lung such as stretching, bioengineers are developing innovative microsystems called “lungs-on-a-chip”, in which the location and mechanical force placed on each cell are painstakingly defined to model both the small airway and the stretching occurring in the alveolar compartment. In the alveolar lung-on-a chip, structural, functional, and mechanical elements of the interface between the alveoli and the capillary network were modeled by microfabricating a microfluidic device that contains two channels separated by a thin flexible membrane coated with ECM proteins. Vascular endothelial cells were cultured in one chamber, and alveolar epithelial cells that could also be exposed to air were cultured in the other. Controlled regulation of membrane stretching and fluid flows through the vascular chamber allowed studying the passage of materials, nutrients or pathogens from the alveolar region to the vascular compartment. These model systems are the only systems to incorporate controlled mechanical stretching and liquid flow control to simulate physiological function at the organ level [85–87]*.* Once again, all of these developments are currently being carried out in human models, but they should also benefit other mammal species.

## Conclusions

Organoids are three-dimensional multicellular structure that reproduces the microanatomy of life-size lungs in vitro. They can be developed using different source of stem cells (embryonic, iPS, adult) and they provide a realistic, animal testing-free method to study tissue and organ development. One of the most common applications of lung organoids is to study how cells respond to infections. And another growing field is the study of respiratory diseases resulting from genetic modifications or physiological alterations. To date, these new efficient in vitro models are mainly developed in human, but efforts must be made to ensure that they can benefit to domestic animals and bring new tools in line with the species of interest. To conclude, the array of different organoids that can be used to model various aspects of lung development, homeostasis, regeneration and disease represents an exciting new avenue for pursuing remaining questions in the lung biology of various species of interest.
